# Post-stroke infection: A systematic review and meta-analysis

**DOI:** 10.1186/1471-2377-11-110

**Published:** 2011-09-20

**Authors:** Willeke F Westendorp, Paul J Nederkoorn, Jan-Dirk Vermeij, Marcel G Dijkgraaf, Diederik van de Beek

**Affiliations:** 1 Department of Neurology, Academic Medical Center, Amsterdam, the Netherlands; 2 Clinical Research Unit, Academic Medical Center, Amsterdam, the Netherlands; 3 Center of Infection and Immunity (CINIMA), Academic Medical Center, Amsterdam, the Netherlands

## Abstract

**Background:**

**s**troke is the main cause of disability in high-income countries, and ranks second as a cause of death worldwide. Patients with acute stroke are at risk for infections, but reported post-stroke infection rates vary considerably. We performed a systematic review and meta-analysis to estimate the pooled post-stroke infection rate and its effect on outcome.

**Methods:**

MEDLINE and EMBASE were searched for studies on post-stroke infection. Cohort studies and randomized clinical trials were included when post-stroke infection rate was reported. Rates of infection were pooled after assessment of heterogeneity. Associations between population- and study characteristics and infection rates were quantified. Finally, we reviewed the association between infection and outcome.

**Results:**

87 studies were included involving 137817 patients. 8 studies were restricted to patients admitted on the intensive care unit (ICU). There was significant heterogeneity between studies (P < 0.001, I^2 ^= 97%). The overall pooled infection rate was 30% (24-36%); rates of pneumonia and urinary tract infection were 10% (95% confidence interval [CI] 9-10%) and 10% (95%CI 9-12%). For ICU studies, these rates were substantially higher with 45% (95% CI 38-52%), 28% (95%CI 18-38%) and 20% (95%CI 0-40%). Rates of pneumonia were higher in studies that specifically evaluated infections and in consecutive studies. Studies including older patients or more females reported higher rates of urinary tract infection. Pneumonia was significantly associated with death (odds ratio 3.62 (95%CI 2.80-4.68).

**Conclusions:**

Infection complicated acute stroke in 30% of patients. Rates of pneumonia and urinary tract infection after stroke were 10%. Pneumonia was associated with death. Our study stresses the need to prevent infections in patients with stroke.

## Background

Infection is a common complication in the acute phase after stroke. Reported infection rates after stroke vary considerably, ranging 5-65%. Differences in patient populations, study design and definition of infection may account for these large variations in post stroke infection rates [1]. However, a reliable pooled estimate of the infection rate in patients with stroke is lacking. Pneumonia is the most common post-stroke infection and been associated with a relative risk of 3.0 for mortality in a study including 14293 patients with stroke [2]. Consequently, new treatment strategies, *i.e*. preventive antibiotics, are currently under investigation [3]. In this systematic review and meta-analysis we calculated the pooled post-stroke infection rates, identified study and population characteristics associated with infection, and estimated the impact of pneumonia on outcome after stroke.

## Methods

### Selection of studies

Cohort studies and randomized clinical trials (RCT) on ischemic or hemorrhagic stroke with reported rates of infections in the acute phase were included. In- and exclusion criteria are listed in Additional File 1: Table S1. A literature search in MEDLINE (1950 to present) and EMBASE (1980 to present) was performed without language restrictions (Additional File 1: Table S2). Cross references were checked and experts in the field were consulted. Infection was defined according to the criteria used in included studies. Studies including a small subgroup of patients, *i.e*., those restricted to patients with dysphagia, were excluded to minimize selection bias. If two publications described one similar patient group data was used only once; this occurred three times [4-9]. If studies reported data of different treatment arms separately, these were also included as separate groups in our analysis [10,11]. Treatment arms in randomized controlled trials on preventive antibiotic therapy were excluded, since this is likely to influence infection rate and is not part of standard stroke care [12].

### Data extraction

Two independent observers (WFW and JDV) extracted data from selected articles according to predefined definitions. Disagreement was resolved by discussion.

Overall infection rate and rate of pneumonia and urinary tract infection - the most common post-stroke infections - were extracted [13]. When percentages were given, these were converted into absolute numbers. We did not calculate an overall infection rate in each study by adding data on different infections, because of the possibility of two infections occurring in one patient.

We also extracted the study characteristics prospective design, consecutive enrollment, type of stroke, study aim on infection and observation period. Population characteristics extracted were income country, age, gender, ICU study, infarction or bleeding, stroke severity, lowered consciousness, dysphagia and urinary incontinence/retention. Definitions can be found in Additional File 1: Table S3.

### Analyses

For each study we calculated the proportion of overall infection, pneumonia and urinary tract infection. Next, these proportions were pooled using Review Manager to obtain one estimate for each infection. A fixed or random effects model was chosen after tests of heterogeneity (heterogeneity was defined with p-value < 0.05). Subgroup analysis was performed on Intensive Care Unit (ICU) *vs*. non-ICU studies. Subsequently, we performed univariate analyses investigating the association between population and study characteristics and the pooled proportion of infection, pneumonia and urinary tract infection. Pearson or Spearman correlation, Students T-Test, Mann-Whitney U Test or 1-way ANOVA were used when appropriate. Characteristics with a p-value < 0.10 were included in a multivariate regression analysis. To transform the proportion of pneumonia and urinary tract infection in a normally distributed variable we used arc sin-square root and square root transformations. Review Manager 5 and SPSS (version 16.0) were used for the statistical analyses.

## Results

### Included studies

**Figure 1:**
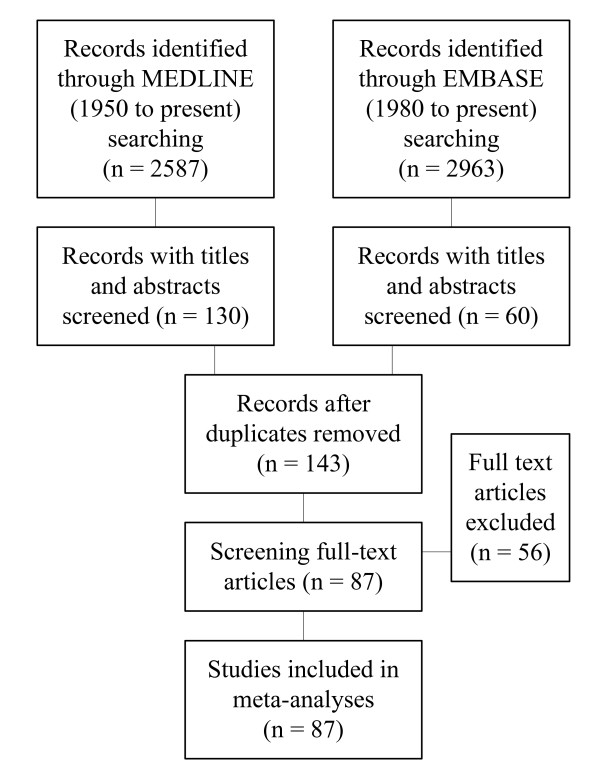
**Flowchart of study selection**.

Figure 1 summarizes the study selection process. 87 studies were included involving 137817 patients. 8 studies included patients admitted on the ICU and 79 studies included patients admitted on stroke-unit, medium care facility or ward (non-ICU studies). Reasons for exclusion are noted in Additional File 1: Table S4. Not all characteristics could be extracted from all studies: age was reported in 77 studies; gender in 80; observation period in 73; stroke severity in 21; lowered consciousness in 25; urinary incontinence/retention in 6; and dysphagia in 26 studies. All extracted data is shown Additional File 2: Table S1.

### Pooled infection rates

Infection rates were evaluated in all 87 studies (Table 1). Pooled values were calculated in a random effects model because of significant heterogeneity between studies (P < 0.001, I^2 ^= 97%). The overall pooled infection rate was 30% (95%CI 24-36%). The pooled pneumonia rate was 10% (95%CI 9-10%) and of urinary tract infection 10% (95%CI 9-12%). In ICU studies, overall infection rate was 45% (95%CI 38-52%) and rates of pneumonia and urinary tract infection were 28% (95%CI 18-38%) and 20% (95%CI 0-40%). In non-ICU studies, overall infection rate was 28% (95%CI 22-34%) and rates of pneumonia and urinary tract infection were 9% (95%CI 9-10%) and 10% (95%CI 8-11%).

**Table 1:** Pooled infection rates in 137817 patients with stroke

	All studies	ICU studies	Non-ICU studies
**No. of included studies**	87	8	79
**No. of evaluated patients**	137817	871	136946
			
**Infection rates**	**% (95%CI)**	**% (95%CI)**	**% (95%CI)**

**All infections**	30 (24-36)	45% (38-52%)	28% (22-34%)
**Pneumonia**	10 (9-10)	28% (18-38%)	9% (9-10%)
**Urinary tract infection**	10 (9-12)	20% (0-40%)	10% (8-11%)

Footnote Table 1: CI denotes confidence interval

### Associations with population- and study characteristics

Associations between population and study characteristics with infection rates are noted in Table 2 and 3. In univariate analysis, infection rate was higher in ICU-studies (*P = 0.05) *and in studies with a longer observation period (*P = 0.03)*; a trend towards significance was seen for lowered consciousness (*P = 0.06)*. We did not perform multivariate analysis for infection, because few studies reported all 3 variables. In the subgroup of non-ICU studies, an even stronger association was seen for observation period (*P *= *0.003)*.

**Table 2:** Univariate analysis between study or population characteristics and reported infection rates

Study/population characteristic*	No. of studies	No. of evaluated patients	P-value
*Study design*			
Prospective design	18	9174	0.82
Consecutive enrollment	18	9174	0.42
Study aim on infection	18	9174	0.89
Observation period	17	8844	0.03
*Population characteristics*			
Income country	18	9174	0.94
Age	18	9174	0.35
Gender	18	9174	0.86
ICU population	18	9174	0.05
Infarction of bleeding	18	9174	0.15
Stroke severity (NIHSS)	5	1818	0.14
Lowered consciousness	6	2365	0.06
Dysphagia	6	395	0.97

Footnote Table 2: definitions of characteristics can be found in Additional File 1: Table S3

**Table 3:** Univariate analysis between study or population characteristics and reported pneumonia rates

Study/population characteristic*	No. of studies	No. of evaluated patients	P-value
*Study design*			
Prospective design	87	137779	0.02
Consecutive enrollment	87	137779	0.005
Study aim on infection	87	137779	0.004
Observation period	73	102280	0.37
*Population characteristics*			
Income country	87	137779	0.23
Age	77	118755	0.30
Gender	80	132750	0.76
ICU population	87	137779	0.001
Infarction of bleeding	87	137779	0.13
Stroke severity (NIHSS)	20	31493	0.01
Lowered consciousness	25	52939	0.001
Dysphagia	26	17937	0.19

Footnotes Table 3: *definitions of characteristics can be found in Additional File 1: Table S3

The rate of pneumonia was higher in ICU studies (*P *= *0.001)*, prospective studies *(P = 0.02)*, studies that specifically evaluated infections *(P = 0.004)*, studies with consecutive enrollment (*P *= *0.005)*, studies with a higher stroke severity (*P *= *0.01) *and studies with higher proportions of patients with a lowered consciousness (*P *= *0.001)*. Stroke severity was closely related to ICU-study (Mann-Whitney U, Z -2,154, *P = 0.02*). In multivariate analysis pneumonia rate was associated with study characteristics: ICU studies (B 0.207, standard error (se) 0.042, *P *<*0.01*), study aim on infection (B 0.062, se 0.026, *P *= *0.02*), consecutive enrollment (B 0.058, se 0.024, *P *= *0.02*). Stroke severity and lowered consciousness were excluded from this multivariate analysis since these characteristics were only reported in a relatively small number of studies. In non-ICU studies, rate of pneumonia was also higher in studies that specifically evaluated infections (B 0.06, se 0.025, *P *= *0.02*) and studies with consecutive enrollment (B 0.065, se 0.022, *P *= *0.005*) in multivariate analysis. No associations were found between age (*P = 0.18) *or dysphagia (*P = 0.16) *and pneumonia rate.

The rate of urinary tract infections was higher in studies with a higher stroke severity (*P = 0.01)*, a lower proportion of male patients (*P *= *0.005)*, prospective studies (*P = 0.02) *and in studies with a longer observation period (*P = 0.10)*. A trend towards significance was seen for studies with a higher mean age (*P = 0.08)*. In multivariate analysis, advanced age was independently associated with urinary tract infection (B 0.008, se 0.004, *P *= *0.04*). Gender was associated with urinary tract infection, but only in a sub analysis of non-ICU studies (B -0,341, se 0.164, *P *= *0.04*).

### Outcome

**Figure 2:**
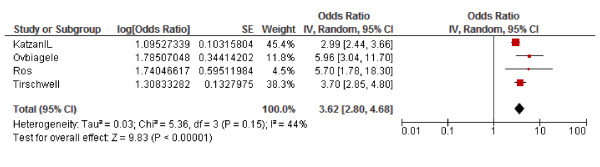
**Pooled odds ratios: effect of pneumonia on in-hospital mortality**.

To estimate the effect of post-stroke infection on outcome we pooled the mortality rates in patients with and without infection of the studies reporting these rates. Of all patients with an infection, 48% died vs. 18% of patients without infection (N = 1839; OR 2.08, 95% CI 1.63 - 2.67). Mortality rates were also higher in patients with pneumonia (26% vs. 5%, N = 16433; OR 5.58, 95% CI 4.76, 6.55) and a little higher in patients with urinary tract infection (12% vs. 10%, N = 2528; OR 1.12, 95% CI 0.76, 1.66). Since this data was not corrected for potential confounders, we also estimated the effect of pneumonia on death by pooling corrected odds ratios (OR) in a random effects model. This meta-analysis included four studies (19971 patients) and resulted in an OR in-hospital mortality of 3.62 (95% CI 2.80-4.68) (Figure 2). Five studies reported corrected OR of pneumonia for unfavorable outcome (Table 4); meta-analysis was not performed because of differences in outcome measure and follow-up. These studies showed an increased risk of unfavorable outcome in post-stroke patients with pneumonia. Urinary tract infection had no association with death according to four studies reporting on this [14-17]. Three studies evaluated the effect on functional outcome for urinary tract infection, two studies showed an association with poor outcome in multivariate analysis [14,18,19]. OR's for unfavorable functional outcome were 2.72 (95% CI 4.73-11.84) and 3.1 (95% CI 1.6-5.9).

**Table 4:** Effect of pneumonia on functional outcome

Study	No. of patients	Outcome (modified Rankin Scale)	OR (95% CI)	Correction for confounders
Vermeij et al [1]	521	> 2 at discharge > 2 at 1 year	9.5 (1.7-52) 10.95 (2.2-46)	Yes
Hong et al [18]	1254	3-6 at 3 months	4.44 (2.20-8.99)	Yes
Huang et al [46]	66	< 3 at 1 month	0.50 (0.38-0.64)	No
Ovbiagele [16]	663	0-3 at discharge	0.16 (0.09-0.29)	Yes
Aslanyan et al [14]	1455	≥ 2 at 3 months	3.4 (1.4-8.3)	Yes

## Discussion

This meta-analysis shows that infections commonly complicate the acute phase after stroke. The pooled overall infection rate was 30% and pneumonia and urinary tract infections occurred each in 10% of patients. Previous studies showed a wide range of post-stroke infection rates, from 5%- 65% for infections, 1%-33% for pneumonia, and 2%-27% for urinary tract infection [1,20]. This meta-analysis included a large number of patients and therefore provides a reliable estimate of infection after stroke.

We identified several study and population characteristics that were associated with infection rate. Given the prevalence of pneumonia and urinary tract infection in general wards in Dutch hospitals - 1.1% and 1.7% respectively - our findings confirm that infection rate in patients in the acute phase of stroke is high [21]. This increased vulnerability of patients in the acute phase of stroke for infections can be attributed to different factors.

First, infection was associated with study characteristics. Studies aimed on infection and those with consecutive enrolment were associated with higher infection rate. Possibly, these studies benefit of more rigorous detection of infection. Rate of infection was also higher in studies with a longer observation time. In a prospective observational study, most post stroke infections occurred within three days of hospital admission [1]. Nevertheless, 25% of infections occurred after these three days. Also, most urinary tract infections occur after 48 hours, i.e. most UTI's are hospital acquired, and therefore it seems logical that a longer observation time yielded a higher urinary tract infection rate in our study [17]. The absence of an association between observation time and pneumonia is not surprising. Pneumonia is mostly diagnosed within the first days following a stroke, both in studies performed on general wards as well as ICU's [1,13,22-24].

Microbiologic data of patients with post-stroke pneumonia shows a pattern of mostly early onset nosocomial pneumonia, or a community acquired aspiration syndrome. *Staphylococcus aureus *and gram-negative bacteria such as *Klebsiella pneumoniae*, *Pseudomonas aeruginosa, Escherichia coli *or *Enterobacter spp*. were commonly identified; also *Streptococcus species *are occasionally found. Gram-negative bacteria and *Staphylococcus aureus *are known to cause pneumonia by aspiration of endogenous material from the colonized oropharynx [25,26]. These pathogens are often seen in nosocomial infections [27]. *Streptococcus species *is still the most detected pathogen in community acquired pneumonia [28]. In stroke patients, it could be a cause of 'community acquired aspiration pneumonia', with aspiration occurring at the stroke ictus [29]. Often, no causative organism is detected in post-stroke pneumonia. Yield of cultures is usually not very high in patients with pneumonia; collection of material could be even more difficult in stroke patients due to neurologic deficit or lowered level of consciousness. Also, some cases of suspected pneumonia could in fact be a non-infectious aspiration pneumonitis, or the infection could be caused by anaerobic bacteria that require special culturing techniques. However, the role of anaerobic bacteria in the development of pneumonia is unclear [29,30].

Second, infection rate was associated with the patients' clinical condition. Studies including patients with a higher stroke severity or lower levels of consciousness showed higher infection rates, in particular for pneumonia. This effect corresponds with previous studies that often report both characteristics as risk factors for pneumonia [14,31,32]. Risk for aspiration is increased in these patients due to the absence of protective reflexes, and this risk is related to the degree of consciousness impairment [33]. Most stroke related pneumonias are believed to result from dysphagia and subsequent aspiration of oropharyngeal material or gastric content. A systematic review by Martino et al showed that dysphagia occurs in 37- 78% of stroke patients and increases the risk for pneumonia 3-fold and 11-fold in patients with confirmed aspiration [34]. However, up to half of patients with post stroke pneumonia do not aspirate, which implies that also other mechanisms are involved, *e.g*., stroke-induced immunodepression which is discussed below [2].

We also found higher infection rates in ICU studies. Patients admitted to an ICU generally suffer from more severe strokes and also the frequency of invasive procedures is higher [15,24,32,35]. The use of invasive procedures - i.e. urinary catheterization or mechanical ventilation in ICU patients - increase infection risk by facilitating the entry of a pathogen [17,35].

We identified a higher age and female sex as risk factors for urinary tract infection. Both of these characteristics have previously been reported as risk factors for urinary tract infection [16,17]. Studies frequently report urinary catheterization as an important risk factor; we could not investigate this association because this characteristic was mostly not reported [17]. Advanced age as risk factor for post-stroke infection has been reported previously [16].

In addition to above mentioned characteristics, acute stroke may lead to stroke-induced immunodepression, a systemic anti-inflammatory response that is related to susceptibility to infection [20,36,37]. This anti-inflammatory response was found in different clinical studies in acute stroke patients, and includes an excessive counter-inflammatory cytokine responses and impairments in cell-mediated immunity [38]. Certain features of this response - *i.e*., reduced lymphocyte count, delay in the recovery of T-lymphocyte loss - were more pronounced in patients developing post stroke infections [37,39]. These results suggest that immunological changes could facilitate infection in acute stroke.

Outcome is affected by post-stroke infections, as shown in our review. Pneumonia and urinary tract infection both increase the risk for unfavorable outcome and pneumonia is associated with mortality with an OR of 3.62. Infections could affect outcome in several ways. Firstly, they lead to immobilization, general frailty and a delay in rehabilitation due to prolonged hospital stay [1,40]. More importantly, immunological effects of infections could worsen outcome. Evidence from experimental studies suggests that infection promotes antigen presentation and autoimmunity against the brain [41]. However, the evidence on this topic is scarce and the exact pathofysiology remains to be investigated.

Our systematic review has limitations. First, results are limited by publication bias. Data was derived from randomized controlled trials, cohort studies and stroke registries; infection rate could differ in hospitals without stroke research or complication registries. Second, included studies were heterogeneous in definition of infection, which was based on clinical grounds and in some cases not described. A standardized definition for infection - as described by the Centers for Disease Control and Prevention [42] - is preferred since stricter criteria could permit identification of fewer infections. Next, not all relevant characteristics - for example use of antibiotics, differences in primary stroke care or use of a urinary catheter - could be evaluated, due to lack of data in included studies. Some of these characteristics have previously been described as a risk factor for infection and could have confounded our results. Thirdly, for some characteristics data was lacking in many studies. Surprisingly, no association was found between age or dysphagia and pneumonia rate, often described risk factors for pneumonia [24,31,32,35,43,44]. Dysphagia was not reported in all studies, which was a limiting factor in the analysis. Lack of these significant associations could be due to few studies reporting adequate information on all these characteristics and therefore the analyses have low explanatory power. Finally, many of the characteristics act on patient level, but studies report aggregate data (*i.e*., mean age of study population) also reducing explanatory power. Due to these limitations - most importantly the heterogeneity in definition of infections - results of this review need to be interpreted with caution.

Our review shows the potential of strategies aiming to prevent infections in patients with stroke. Some of these strategies - *i.e.*, prevention of aspiration and reduction of urinary catheterisation - are incorporated in stroke unit care, which reduces the risk of death after stroke through the prevention of infections in particular [45]. Infections can also be prevented by use of preventive antibiotic therapy, as shown in a recent meta-analysis. This meta-analysis did not establish a reduction in mortality, however, included studies were small and heterogeneous and functional outcome was not evaluated [12]. Also, limited data was reported on the effect on antibiotic resistance. Currently, the use of preventive antibiotics in stroke, and the effect of this therapy on antibiotic resistance, is investigated in a large randomized controlled trial with functional endpoint [3]. This trial will be able to establish whether preventive antibiotic treatment is an effective strategy to prevent infection and its adverse effect on outcome in patients in the acute phase of stroke.

## Conclusions

Results of this meta-analysis show an overall infection rate in the acute phase of stroke of 30%. Rates of pneumonia and urinary tract infection were both 10%. Infection rates are related with study characteristics and the patients' clinical condition, *e.g*., age, gender, stroke severity, level of consciousness and whether a patient is admitted on ICU. Pneumonia is an independent risk factor for unfavorable outcome and death after stroke. Our data stress the need of interventions to prevent infections in patients with stroke.

## Competing interests

The authors declare that they have no competing interests.

## Authors' contributions

WFW participated in the conceptualization and design of the review, performed the selection of studies, data-extraction and -analysis, and drafted the review. PJN and DvdB were involved in the conceptualization and design of the review, and the data analysis. JDV participated in the selection of studies and data-extraction. MGD carried out the statistical analysis and interpretation of data. All authors participated in revising the manuscript and the final approval of the manuscript.

## Sources of funding

This work was supported by the Netherlands Organisation for Health Research and Development (ZonMW): 171002302 and the Netherlands Heart Foundation (Hartstichting): 2009B095. DvdB is supported by grants from the Netherlands Organization for Health Research and Development (ZonMw; NWOVeni grant 2006 [916.76.023], NWO-Vidi grant 2010 [016.116.358]) and the Academic Medical Center (AMC Fellowship 2008).

## Pre-publication history

The pre-publication history for this paper can be accessed here:

http://www.biomedcentral.com/1471-2377/11/110/prepub

## Supplementary Material

Additional file 1 **selection of studies, data-extraction and excluded studies**. **Table S1**: in- and exclusioncriteria. **Table S2**: synonyms for MEDLINE and EMBASE search. **Table S3**: definitions of characteristics. **Table S4**: reason for exclusion on full-text. Click here for file

Additional file 2 **data extracted from included studies**. **Table S1**: extracted data. Click here for file
