# Supplementary material for: Post-stroke infection: A systematic review and meta-analysis
Source: BMC Neurol. 2011 Sep 20;11:110. doi: 10.1186/1471-2377-11-110 (PMC3185266; doi:10.1186/1471-2377-11-110)
Supplement: Additional file 1 — selection of studies, data-extraction and excluded studies. Table S1: in- and exclusioncriteria. Table S2: synonyms for MEDLINE and EMBASE search. Table S3: definitions of characteristics. Table S4: reason for exclusion on full-text. [file 1471-2377-11-110-S1.PDF]

ADDITIONAL FILE 1

Table 1: *in- and exclusioncriteria*

**Inclusion criteria**	**Exclusion criteria**
Adult patients with acute stroke	Rehabilitation setting
Cohort study or<br>Randomized Controlled Trial	Reviews, case reports,<br>studies with < 25 patients
Post stroke infection rate reported	Infection preceding stroke of after discharge
English, German, French, Spanish language	Study performed solely on<br>subgroup of patients
Full text available	

Table 2: *synonyms for MEDLINE and EMBASE search*

**Synonyms for MEDLINE Search**
(cerebrovascular disorders/ or exp basal ganglia cerebrovascular disease/ or exp brain ischemia/ or stroke/ or exp brain infarction/ or hypoxia-ischemia, brain/ or exp intracranial arterial diseases/ or exp "Intracranial Embolism and Thrombosis"/ or exp intracranial hemorrhages/ or (stroke or poststroke or post-stroke or cerebrovasc$ or brain vasc$ or cerebral vasc$ or cva$ or apoplex$ or isch?emi$ attack$ or tia$1 or neurologic$ deficit$).tw. or ((brain$ or cerebr$ or cerebell$ or cortical or vertebrobasilar or hemispher$ or intracran$ or intracerebral or infratentorial or supratentorial or MCA or anterior circulation or posterior circulation or basal ganglia) adj5 (isch?emi$ or infarct$ or emboli$ or occlus$ or hypox$ or obstruction or vasculopathy)).tw. or ((lacunar or cortical) adj5 infarct$).tw. or ((brain$ or cerebr$ or cerebell$ or intracerebral or intracran$ or parenchymal or intraventricular or infratentorial or supratentorial or basal gangli$ or subarachnoid or putaminal or putamen or posterior fossa) adj5 (haemorrhage$ or hemorrhage$ or haematoma$ or hematoma$ or bleed$)).tw. or ((brain or intracranial or basal ganglia or lenticulostriate) adj5 (vascular adj5 (disease$ or disorder or event))).tw. or ((isch?emic or apoplectic) adj5 (event or events or insult or attack$)).tw. or ((intracranial or cerebral art$ or basilar art$ or vertebral art$ or vertebrobasilar or vertebral basilar) adj5 (stenosis or ischemia or insufficiency or arterioscleró$ or atheroscleró$ or occlus$)).tw. or ((unilateral or visual or hemispatial or attentional or spatial) adj5 neglect).tw.) not (stroke adj volume).tw. not ((exp child/ or exp infant/) not ((exp child/ or exp infant/) and exp adult/))
AND
exp incidence/ or exp prevalence/ or exp epidemiology/ or occurrence.tw. or frequenc$.tw. or incidence.tw. or prevalence.tw. or exp Cross-Sectional Studies/ or exp Health Surveys/ or (health adj3 survey$).ti,ab. or (population adj3 based).ti,ab. or outcome.ti,ab. or prognos$.ti,ab or (follow adj 2 up adj2 stud$).ti,ab or mortality.ti,ab or predict$.ti,ab
AND
exp Urinary Tract Infections/ or uti.ti,ab or exp Cystitis/ or Cystitis.ti,ab. or exp Pneumonia/ or Pneumonia.ti,ab. or rti.ti,ab or fever.ti,ab or (stroke adj4 complication$).ti,ab or ((pulmonary or lung or airway or chest or respiratory or urinary) adj2 (inflammation$ or infection$)).ti,ab
**Mesh terms for EMBASE search**
exp stroke/ OR exp brain ischemia/ OR exp intracranial hemorrhage/
AND
exp pneumonia/ OR exp respiratory tract infection/ OR exp cystitis/ OR exp urinary tract infections/

Table 3: *definitions of characteristics*

Study/population characteristic	Definition
Study design	
Prospective design	Whether the study design was prospective
Consecutive enrollment	Whether patients were included consecutively. When not reported, this was regarded as not consecutive
Study aim on infection	Whether post stroke infection was the primary focus of the study or not
Observation period	The time in days in which occurrence of infection was scored (length of stay or a predetermined interval; when occurrence of infection was scored during different time periods, the shortest interval was used)
Population characteristics	
Income country	Economies are divided into high (= 0) and low (= 1) income, according to 2008 GNI per capital (low income: $975 or less and lower middle income, $976 - $3,855, high: upper middle income, $3,856 - $11,905 and high income, $11,906 or more) (worldbank.org)
Age	Mean age of included patients *
Gender	Number of male patients **
ICU study	Whether the study was performed on an ICU or not
Infarction or bleeding	Ischemic stroke (IS), hemorrhagic stroke (HS), or both (S)
Stroke severity (NIHSS)	Mean NIHSS-score on admission (other scales were not used, neither were median values) *
Lowered consciousness	Number of patients with a reduced consciousness on admission
Dysphagia	Number of patients with dysphagia on admission (according to the definition used in the study)
Urinary incontinence/retention	Number of patients with urinary incontinence or urinary retention on admission (as defined in the study)
Outcome	
Infection	Number of patients with any infection, defined on criteria used in study** (only used when exact number was given and not calculated by adding numbers of different infections, because 2 infections could have occurred in 1 patient)
Pneumonia	Number of patients with pneumonia, defined on criteria used in study*
Urinary tract infection	Number of patients with urinary tract infection, defined on criteria used in study**

* when mean values for different groups were given, a general mean was calculated

** when percentages were given, numbers were calculated

Table 4: *reason for exclusion on full-text*

**Infection rate not reported** Bamford et al, 1990; Broessner G et al 2009; Christensen et al 2009; Czlonkowska et al, 2002; Dumas et al 1994; Elkind MS et al 2004/2007; Gray et al, 2007; Lodder et al 2006; MacWalter et al 1995; Poungvarin et al 2006; Reggiani M et al, 2009; Ryglewicz D; Silver et al 1984; Sweileh WM et al 2006-2007; Vernino et al 2003; Marti-Vilalta et al: Barcelona stroke registry, 1999.
**Rehabilitationsetting after acute phase of stroke** Addington et al 1999; Arai et al 2004; Culebras et al, 2007; Doshi et al 2003; Dromerick et al 2003; Ersoz 2007;Falsetti et al 2009; Harada et al, 2006; Kalra et al, 1995; Langhorne et al, 2000; Lipson et al, 2005; Luk et al, 2006; Marciniak et al; Meng et al, 2000; Rocco A et al 2007; Roth EJ 2001; Werner et al 1998.
**No fulltext available** (after request in medical library and attempts to contact the author by e-mail) Gonzales MJ et al 1995; Lee HC et al, 2008; Suwanwela et al, 2007; Morales-Ortiz et al, 2001; Werner et al 1998 (1572); Roth et al, 2007; Huang et al, 2005
**Small study** Chang KC et al ; Knoll T et al 2002
**Different domain (e.g. study on subgroup, or SAH included)** Commichau C et al 2003; Farooq et al, 2008; Kalra et al 2000 ; Kim H et al 2000; Ali M et al, 2009; Ding R et al, 2000
**Language** Aslan et al, 2007
**Similar patient population** Dziedzic et al, 2009; Lees et al, 2006; Schwab S et al,1999
