# Supplementary material for: Post-stroke infection: A systematic review and meta-analysis
Source: BMC Neurol. 2011 Sep 20;11:110. doi: 10.1186/1471-2377-11-110 (PMC3185266; doi:10.1186/1471-2377-11-110)
Supplement: Additional file 2 — data extracted from included studies. Table S1: extracted data. [file 1471-2377-11-110-S2.PDF]

ADDITIONAL FILE 2

Table 1
Extracted data

	Name, Year	Number of Patients	Age	Infection rate	Pneumonia rate	UTI rate	High (0) /low (1) income country	Study Design (prospective =0, retrospective=1)	Consecutive study (=1)	Domain	Gender	ICU study(=1)	Time of observation	Stroke severity	Dysphagia	Study aim (1 = study on infection)	Lowered consciousness	Disorders of urinary tract
1	Minnerup J	591	67,7	237	72	65	0	0	1	S	325	0	14	7	-	1	105	-
2	Pilar Grajales Cuesy, 2010 [1]	255	73,3	-	44	-	0	0	0	IS	102	0	-	-	43	1	143	-
3	Eriksson M, 2009[2]	24633	76	-	988	-	0	1	0	S	12350	0	17	-	-	0	4419	-
4	Den Hertog, 2009 [3]	1400	69,9	-	33	10	0	0	0	S	784	0	-	6	0	0	-	-

5	Langdon, 2009[4]	330	71,1	115	51	52	0	0	0	IS	170	0	30	-	74	1	-	127
6	Levy DE, 2009[5]	500	69,9	170	23	86	0	0	0	S	246	0	90	11	-	0	-	-
7	Saposnik, 2008[6]	3631	72	-	240	-	0	0	1	IS	1895	0	30	-	418	0	483	-
8	Sorbello D., 2009 [7]	71	74,7	-	11	14	0	0	0	S	38	0	90	-	-	0	-	-
9	Stott DJ, 2009 [8] Sellars C, 2007 [9]	412	67,9	-	78	65	0	0	1	S	205	0	90	-	-	1	-	-
10	Vermeij, 2009 [10]	521	70,5	78	39	23	0	0	1	IS	281	0	7	-	-	1	58	-
11	Gargano, 2008 [11]	2566	68,4	-	146	204	0	1	0	S	1185	0	-	-	-	0	-	-
12	Harms, 2008 [12]	40	72,7	13	8	5	0	0	0	IS	29	0	11	-	27	1	-	-

13	Hong, 2008 [13]	1254	66,5	-	151	86	0	0	1	IS	703	0	28	4	-	0	-	-
14	Horner, 2008 [14]	13831	70,5	-	763	680	0	1	0	IS	7161	0	90	6	-	0	-	-
15	Huang, 2008 [15]	66	67,6	-	6	2	1	0	1	IS	51	0	30	-	-	0		-
16	Indredavik, 2008 [16]	489	77,2	-	55	78	0	0	1	S	233	0	7	-	-	0	104	-
17	Naidech, 2008 [17]	56	63,9	-	9	-	0	0	1	HS	29	1	8	-	-	0	43	-
18	Navarro JC, 2008 [18]	1153	62	-	95	50	1	0	1	S	666	0	14	-	-	0		49
19	Reid JM, 2008 [19]	2725		-	141	353	0	1	1	S	1414	0	-	-	-	0	-	-

20	Schwarz, 2008 [20]	30	73	27	7	18	0	0	0	IS	30	0	10	15	-	1	-	-
21	Sposato, 2008 [21]	1991	69,4	-	285	-	0	0	1	IS	1100	0	8	-	-	0	-	-
22	Yan, 2008 [22]	137	71	67	63	19	1	1	1	S	84	1	5	-	62	0	80	-
23	Lee M, 2007 [23]	947	69,6	-	104	119	1	0	1	IS	502	0	18	-	-	0	129	-
24	Ros, 2007 [24]	258	74,9	102	24	38	0	0	1	S	126	0	11	13	114	1	-	-
25	Roth, 2007	2457	63,4	-	379	498	0	1	1	S	1214	0	17	9	-	0	-	-
26	Shuaib, 2007 [25]	3195	68,9	-	193	325	0	0	0	IS	1768	0	90	13	NR	0	-	-

27	Sundar U, 2007 [26]	184	-	-	29	8	1	0	1	IS	-	0	14	-	43	0	-	-
28	Walter U, 2007 [27]	236	69,8	-	51	-	0	0	0	IS	124	1	5	-	69	1	65	-
29	Yilmaz, 2007 [28]	171	66	71	44	70	0	0	0	S	90	1	11	-	-	1	-	-
30	Chiu, 2006 [29]	46	79	-	7	18	1	0	1	IS	14	0	17	19	-	0	-	-
31	Dziedzic, 2006 [30]	833	69,0	-	89	-	0	1	1	IS	389	0	30	-	-	0	-	-
32	Dziedzic, 2006 [31]	705	69,7	-	74	-	0	1	1	IS	320	0	12	-	-	1	-	-
33	Gosney, 2006 [32]	100	-	-	7	-	0	0	0	S	106	0	21	-	33	1	-	-

34	Hassan A, 2006 [33]	443	58	-	102	-	1	1	0	S	-	0	4	-	-	1	-	-
35	Kwan J, 2007 [34]	439	74	73	45	30	0	0	1	S	215	0	5	0	149	1	118	183
36	Kwon HM, 2006 [35]	286	62,8	-	47	-	0	0	1	S	192	0	30	13	96	1	-	-
37	Langdon PC, 2006 [36]	88	74,6	25	16	10	0	0	0	IS	43	0	30	-	58	0	36	-
38	Maramattom, 2006	144	71,3	-	28	-	0	0	1	HS	89	0	6	-	-	0	69	-
39	Matz, 2006[39]	238	71,8	-	33	17	0	0	1	S	109	0	10	5	-	0	-	-
40	Ovbiagele B, 2006 [37]	663		-	66	84	0	1	0	IS	297	0	-	-	-	1	-	-

41	Vargas, 2006 [38]	229	72,6	60	33	13	0	0	1	S	117	0	7	-	-	1	-	-
42	Secades, 2006 [39]	38	70,8	-	-	1	0	0	0	HS	19	0	90	12	-	0	-	-
43	Garbusinski, 2005[40]	148	-	-	27	-	1	0	1	S	73	0		16	90	0	68	-
44	Hanchaiphibool kul	332	62	22	12	13	1	1	0	IS	209	0	3	-	-	0	31	-
45	Hinchey,2005 [41]	2532	70,5	-	114	-	0	0	0	IS	1262	0	6	-		1	-	-
46	Misra, 2005 [42]	141	57,2	-	12	-	1	0	0	HS	101	0	30	-	-	0	-	-
47	Heuschman, 2004 [43]	10800	70	-	648	-	0	1	1	IS	5751	0	11	-	-	0	-	-

48	Field, 2004 [44]	11642	-	-	541	-	0	1	?0	S	5856	0	-	-	-	0	-	-
49	Kwan, 2004 [45]	351	74,5	-	37	25	0	-	1	S	173	0	5	-	149	0	-	-
50	Lees, 2004 [46]	2386	70,3	-	19	-	0	0	0	IS	1277	0	2	-	-	0	-	-
51	Steger, 2004[47]	992	76,2	-	135	142	0	0	1	S	425	0	-	-	-	0	-	-
52	Upadya A, 2004 [48]	55	69,6	-	26	-	0	1	0	S	31	1	16	17	-	1	-	-
53	Aslanyan S, 2003 [49]	1455	70	-	159	146	0	0	0	IS	815	0	7	13	-	1	-	-
54	Broadley, 2003 [50]	149	70	-	7	-	0	0	1	S	88	0	-	-	74	0	18	44

55	FOOD-collaboration, 2003 [51]	3012	73,3	-	367	-	0	0	0	S	1520	0	40	-	732	0	-	-
56	Hamidon BB, 2003 [52]	163	62,2	26	20	6	0	0	0	IS	105	0	3	-	-	1	-	-
57	Hilker, 2003 [53]	124	63,8	-	26	-	0	0	1	S	82	1	8	-	36	1	-	-
58	Katzan IL, 2003 [54]	11286	76,8	-	635	-	0	1	0	S	7888	0		-	-	1	1696	-
59	Lang, 2003 [55]	2030		-	73	40	0	1	0	IS	-	0	5	-	-	0	-	-
60	Martinsson, 2003 [56]	45	67,3	-	6	3	0	0	0	IS	26	0	90	-	-	0	7	-
61	Pittock SJ, 2003 [57]	117	69,9	-	11	4	0	0	1	IS	68	0	14	-	33	0	-	29

62	Spratt, 2002 [58]	257	73	51	26	33	0	0	1	IS	127	0	21	-	-	0	43	-
63	Weimar, 2002 [59]	3866	66,6	-	286	244	0	0	?0	IS	2241	0	7	8	-	0	766	-
64	Evans, 2001 [60]	304	76	-	58	-	0	0	0	S	155	0	90	-	-	0	-	-
65	Kammersgaard LP, 2001 [61]	1156	74,2	225	82	143	0	0	1	S	531	0	3	-	-	1	-	-
66	Koennecke, 2001 [62]	42	70,3	-	2	2	0	0	1	IS	22	1	10	-	-	0	-	-
67	Enlimomab Stroke Trial, 2001 [63]	625	68,9	-	12	-	0	0	0	IS	344	0	5	15	-	0	-	-
68	Schwab, 2001 [64]	50	57	-	24	-	0	0	1	IS	35	1	-	25	-	0	-	-

69	Georgilis K, 1999 [65]	330	72,8	75	33	38	0	1	0	S	184	0	-	-	-	0	-	-
70	Grau AJ, 1999 [66]	119	61	17	10	2	0	0	1	IS	79	0	2	-	-	1	-	-
71	Tirschwell DL, 1999[67]	4757	75	-	338	442	0	1	0	IS	2041	0	8	-	266	0	-	-
72	Hacke, 1998 [68]	37	68,5	-	3	-	0	0	0	IS	21	0	28	-	-	0	-	-
73	Hinds, 1998 [69]	115	74,9	-	27	-	0	0	1	S	51	0	13	-	62	0	22	-
74	Johnston, 1998 [70]	279	69	-	27	30	0	0	0	IS	159	0	90	-	15	0	-	14
75	Newell, 1995 [71]	356	-	-	23	-	0	1	0	IS	-	0	10	-	-	0	-	-

76	Newell, 1997	399	-	-	11	-	0	1	0	IS	-	0	7	-	-	0	-	-
77	Nilsson, 1998 [72]	100	75,4	-	5	-	0	0	1	S	36	0	-	-	14	0	-	-
78	Pinto, 1998 [73]	213	59,2	-	21	37	0	0	1	S	113	0	10	-	-	0	14	-
79	Sala, 1998	187	73,3	-	13	-	0	0	1	S	95	0	10	-	135	0	55	-
80	Schneider, 1998 [75]	32	63	-	10	-	0	0	0	S	14	0		-	-	0	-	-
81	Grotta J, 1997 [76]	721	70,5	-	59	95	0	0	0	IS	-	0	7	15	-	0	-	-
82	Davenport, 1996 [77]	607	73	-	70	98	0	1	1	S	279	0	37	-	-	0	-	-

83	Kidd, 1995 [78]	60	72	-	19	-	0	0	1	S	25	0	14	-	25	1	-	-
84	Webb, 1995 [79]	383	-	-	12	64	0	1	0	S	-	0	10	-	-	0	-	-
85	Webb	303	-	-	5	35	0	1	0	S	-	0	8	-	-	0	-	-
86	Oddersson, [80]	211	74,1	-	11	24	0	0	0	IS	-	0	8	-	-	0	-	-
87	Spitzer, 1988 [81]	63	50,1	-	20	12	0	1	1	HS	31	0	-	-	-	0	-	-
88	Gordon, 1987 [82]	91	-	-	11	-	0	0	1	S	38	0		-	41	0	55	-
89	Przelomski, 1986 [83]	104	71,8	-	13	2	0	0	1	S	55	0	5	-	-	0	-	-

Reference List

1. Cuesy PG, Sotomayor PL, Pina JO. Reduction in the incidence of poststroke nosocomial pneumonia by using the "turn-mob" program. J Stroke Cerebrovasc Dis 2010;19(1):23-28.

2. Eriksson M, Glader EL, Norrving B, Terent A, Stegmayr B. Sex differences in stroke care and outcome in the Swedish national quality register for stroke care. Stroke 2009;40(3):909-914.

3. den Hertog HM, van der Worp HB, van Gemert HM et al. The Paracetamol (Acetaminophen) In Stroke (PAIS) trial: a multicentre, randomised, placebo-controlled, phase III trial. Lancet Neurol 2009;8(5):434-440.

4. Langdon PC, Lee AH, Binns CW. High incidence of respiratory infections in 'nil by mouth' tube-fed acute ischemic stroke patients. Neuroepidemiology 2009;32(2):107-113.

5. Levy DE, del Zoppo GJ, Demaerschalk BM et al. Ancrod in acute ischemic stroke: results of 500 subjects beginning treatment within 6 hours of stroke onset in the ancrod stroke program. Stroke 2009;40(12):3796-3803.

6. Saposnik G, Hill MD, O'Donnell M, Fang J, Hachinski V, Kapral MK. Variables associated with 7-day, 30-day, and 1-year fatality after ischemic stroke. Stroke 2008;39(8):2318-2324.

7. Sorbello D, Dewey HM, Churilov L et al. Very early mobilisation and complications in the first 3 months after stroke: further results from phase II of A Very Early Rehabilitation Trial (AVERT). Cerebrovasc Dis 2009;28(4):378-383.

8. Stott DJ, Falconer A, Miller H, Tilston JC, Langhorne P. Urinary tract infection after stroke. QJM 2009;102(4):243-249.

9. Sellars C, Bowie L, Bagg J et al. Risk factors for chest infection in acute stroke: a prospective cohort study. Stroke 2007;38(8):2284-2291.

10. Vermeij FH, Scholte op Reimer WJ, de MP et al. Stroke-associated infection is an independent risk factor for poor outcome after acute ischemic stroke: data from the Netherlands Stroke Survey. Cerebrovasc Dis 2009;27(5):465-471.

11. Gargano JW, Wehner S, Reeves M. Sex differences in acute stroke care in a statewide stroke registry. Stroke 2008;39(1):24-29.

12. Harms H, Prass K, Meisel C et al. Preventive antibacterial therapy in acute ischemic stroke: a randomized controlled trial. PLoS One 2008;3(5):e2158.

13. Hong KS, Kang DW, Koo JS et al. Impact of neurological and medical complications on 3-month outcomes in acute ischaemic stroke. Eur J Neurol 2008;15(12):1324-1331.

14. Horner S, Niederkorn K, Schnabl S, Fazekas F. [Gender aspects of Ischemic stroke. An analysis of the Austrian Stroke-Unit Registry]. Wien Med Wochenschr 2008;158(15-16):446-452.

15. Huang WY, Weng WC, Chien YY, Wu CL, Peng TI, Chen KH. Predictive factors of outcome and stroke recurrence in patients with unilateral atherosclerosis-related internal carotid artery occlusion. Neurol India 2008;56(2):173-178.

16. Indredavik B, Rohweder G, Naalsund E, Lydersen S. Medical complications in a comprehensive stroke unit and an early supported discharge service. Stroke 2008;39(2):414-420.

17. Naidech AM, Bendok BR, Tamul P et al. Medical complications drive length of stay after brain hemorrhage: a cohort study. Neurocrit Care 2009;10(1):11-19.

18. Jose C Navarro, Ester Bitanga, Nijasri Suwanwela et al. Complication of acute stroke: A study in ten Asian countries. Neurology Asia [13: 33-39]. 2010.
Ref Type: Generic

19. Reid JM, Dai D, Gubitz GJ, Kapral MK, Christian C, Phillips SJ. Gender differences in stroke examined in a 10-year cohort of patients admitted to a Canadian teaching hospital. Stroke 2008;39(4):1090-1095.

20. Schwarz S, Al-Shajlawi F, Sick C, Meairs S, Hennerici MG. Effects of prophylactic antibiotic therapy with mezlocillin plus sulbactam on the incidence and height of fever after severe acute ischemic stroke: the Mannheim infection in stroke study (MISS). Stroke 2008;39(4):1220-1227.

21. Sposato LA, Esnaola MM, Zamora R, Zurru MC, Fustinoni O, Saposnik G. Quality of ischemic stroke care in emerging countries: the Argentinian National Stroke Registry (ReNACer). Stroke 2008;39(11):3036-3041.

22. Yan F, Zhang D, Xu H, Guo H. Risk factors for fever in critically ill patients with acute new-onset stroke. Neurol Res 2008;30(4):394-399.

23. Lee M, Huang WY, Weng HH, Lee JD, Lee TH. First-ever ischemic stroke in very old Asians: clinical features, stroke subtypes, risk factors and outcome. Eur Neurol 2007;58(1):44-48.

24. Ros L, Garcia M, Prat J et al. [Predictors of nosocomial infection in acute stroke. Relation with morbimortality and outcome]. Med Clin (Barc) 2007;128(12):441-447.

25. Shuaib A, Lees KR, Lyden P et al. NXY-059 for the treatment of acute ischemic stroke. N Engl J Med 2007;357(6):562-571.

26. Sundar U, Mehetre R. Etiopathogenesis and predictors of in-hospital morbidity and mortality in posterior circulation strokes--a 2 year registry with concordant comparison with anterior circulation strokes. J Assoc Physicians India 2007;55:846-850.

27. Walter U, Knoblich R, Steinhagen V, Donat M, Benecke R, Kloth A. Predictors of pneumonia in acute stroke patients admitted to a neurological intensive care unit. J Neurol 2007;254(10):1323-1329.

28. Yilmaz GR, Cevik MA, Erdinc FS, Ucler S, Tulek N. The risk factors for infections acquired by cerebral hemorrhage and cerebral infarct patients in a neurology intensive care unit in Turkey. Jpn J Infect Dis 2007;60(2-3):87-91.

29. Chiu EH, Liu CS, Tan TY, Chang KC. Venturi mask adjuvant oxygen therapy in severe acute ischemic stroke. Arch Neurol 2006;63(5):741-744.

30. Dziedzic T, Slowik A, Pera J, Szczudlik A. Beta-blockers reduce the risk of early death in ischemic stroke. J Neurol Sci 2007;252(1):53-56.

31. Dziedzic T, Pera J, Klimkowicz A et al. Serum albumin level and nosocomial pneumonia in stroke patients. Eur J Neurol 2006;13(3):299-301.

32. Gosney M, Martin MV, Wright AE. The role of selective decontamination of the digestive tract in acute stroke. Age Ageing 2006;35(1):42-47.

33. Hassan A, Khealani BA, Shafqat S et al. Stroke-associated pneumonia: microbiological data and outcome. Singapore Med J 2006;47(3):204-207.

34. Kwan J, Hand P. Infection after acute stroke is associated with poor short-term outcome. Acta Neurol Scand 2007;115(5):331-338.

35. Kwon HM, Jeong SW, Lee SH, Yoon BW. The pneumonia score: a simple grading scale for prediction of pneumonia after acute stroke. Am J Infect Control 2006;34(2):64-68.

36. Langdon PC, Lee AH, Binns CW. Dysphagia in acute ischaemic stroke: severity, recovery and relationship to stroke subtype. J Clin Neurosci 2007;14(7):630-634.

37. Ovbiagele B, Hills NK, Saver JL, Johnston SC. Frequency and determinants of pneumonia and urinary tract infection during stroke hospitalization. J Stroke Cerebrovasc Dis 2006;15(5):209-213.

38. Vargas M, Horcajada JP, Obach V et al. Clinical consequences of infection in patients with acute stroke: is it prime time for further antibiotic trials? Stroke 2006;37(2):461-465.

39. Secades JJ, Alvarez-Sabin J, Rubio F, Lozano R, Davalos A, Castillo J. Citicoline in intracerebral haemorrhage: a double-blind, randomized, placebo-controlled, multi-centre pilot study. Cerebrovasc Dis 2006;21(5-6):380-385.

40. Garbusinski JM, van der Sande MA, Bartholome EJ et al. Stroke presentation and outcome in developing countries: a prospective study in the Gambia. Stroke 2005;36(7):1388-1393.

41. Hinchey JA, Shephard T, Furie K, Smith D, Wang D, Tonn S. Formal dysphagia screening protocols prevent pneumonia. Stroke 2005;36(9):1972-1976.

42. Misra UK, Kalita J, Pandey S, Mandal SK, Srivastava M. A randomized placebo controlled trial of ranitidine versus sucralfate in patients with spontaneous intracerebral hemorrhage for prevention of gastric hemorrhage. J Neurol Sci 2005;239(1):5-10.

43. Heuschmann PU, Kolominsky-Rabas PL, Misselwitz B et al. Predictors of in-hospital mortality and attributable risks of death after ischemic stroke: the German Stroke Registers Study Group. Arch Intern Med 2004;164(16):1761-1768.

44. Field TS, Green TL, Roy K, Pedersen J, Hill MD. Trends in hospital admission for stroke in Calgary. Can J Neurol Sci 2004;31(3):387-393.

45. Kwan J, Hand P, Dennis M, Sandercock P. Effects of introducing an integrated care pathway in an acute stroke unit. Age Ageing 2004;33(4):362-367.

46. Muir KW, Lees KR, Ford I, Davis S. Magnesium for acute stroke (Intravenous Magnesium Efficacy in Stroke trial): randomised controlled trial. Lancet 2004;363(9407):439-445.

47. Steger C, Pratter A, Martinek-Bregel M et al. Stroke patients with atrial fibrillation have a worse prognosis than patients without: data from the Austrian Stroke registry. Eur Heart J 2004;25(19):1734-1740.

48. Upadya A, Thorevska N, Sena KN, Manthous C, Amoateng-Adjepong Y. Predictors and consequences of pneumonia in critically ill patients with stroke. J Crit Care 2004;19(1):16-22.

49. Aslanyan S, Weir CJ, Diener HC, Kaste M, Lees KR. Pneumonia and urinary tract infection after acute ischaemic stroke: a tertiary analysis of the GAIN International trial. Eur J Neurol 2004;11(1):49-53.

50. Broadley S, Croser D, Cottrell J et al. Predictors of prolonged dysphagia following acute stroke. J Clin Neurosci 2003;10(3):300-305.

51. Poor nutritional status on admission predicts poor outcomes after stroke: observational data from the FOOD trial. Stroke 2003;34(6):1450-1456.

52. Hamidon BB, Raymond AA, Norlinah MI, Jefferelli SB. The predictors of early infection after an acute ischaemic stroke. Singapore Med J 2003;44(7):344-346.

53. Hilker R, Poetter C, Findeisen N et al. Nosocomial pneumonia after acute stroke: implications for neurological intensive care medicine. Stroke 2003;34(4):975-981.

54. Katzan IL, Cebul RD, Husak SH, Dawson NV, Baker DW. The effect of pneumonia on mortality among patients hospitalized for acute stroke. Neurology 2003;60(4):620-625.

55. Lang W, Lalouschek W. [Acute therapy of ischemic stroke]. Wien Med Wochenschr 2003;153(1-2):21-24.

56. Martinsson L, Wahlgren NG. Safety of dexamphetamine in acute ischemic stroke: a randomized, double-blind, controlled dose-escalation trial. Stroke 2003;34(2):475-481.

57. Pittock SJ, Meldrum D, Hardiman O, Thornton J, Brennan P, Moroney JT. The Oxfordshire Community Stroke Project classification: correlation with imaging, associated complications, and prediction of outcome in acute ischemic stroke. J Stroke Cerebrovasc Dis 2003;12(1):1-7.

58. Spratt N, Wang Y, Levi C, Ng K, Evans M, Fisher J. A prospective study of predictors of prolonged hospital stay and disability after stroke. J Clin Neurosci 2003;10(6):665-669.

59. Weimar C, Roth MP, Zillessen G et al. Complications following acute ischemic stroke. Eur Neurol 2002;48(3):133-140.

60. Evans A, Perez I, Harraf F et al. Can differences in management processes explain different outcomes between stroke unit and stroke-team care? Lancet 2001;358(9293):1586-1592.

61. Kammersgaard LP, Jorgensen HS, Reith J et al. Early infection and prognosis after acute stroke: the Copenhagen Stroke Study. J Stroke Cerebrovasc Dis 2001;10(5):217-221.

62. Koennecke HC, Leistner S. Prophylactic antipyretic treatment with acetaminophen in acute ischemic stroke: a pilot study. Neurology 2001;57(12):2301-2303.

63. Use of anti-ICAM-1 therapy in ischemic stroke: results of the Enlimomab Acute Stroke Trial. Neurology 2001;57(8):1428-1434.

64. Schwab S, Georgiadis D, Berrouschot J, Schellinger PD, Graffagnino C, Mayer SA. Feasibility and safety of moderate hypothermia after massive hemispheric infarction. Stroke 2001;32(9):2033-2035.

65. Georgilis K, Plomaritoglou A, Dafni U, Bassiakos Y, Vemmos K. Aetiology of fever in patients with acute stroke. J Intern Med 1999;246(2):203-209.

66. Grau AJ, Buggle F, Schnitzler P, Spiel M, Lichy C, Hacke W. Fever and infection early after ischemic stroke. J Neurol Sci 1999;171(2):115-120.

67. Tirschwell DL, Kukull WA, Longstreth WT, Jr. Medical complications of ischemic stroke and length of hospital stay: experience in Seattle, Washington. J Stroke Cerebrovasc Dis 1999;8(5):336-343.

68. Hacke W, Donnan G, Fieschi C et al. Association of outcome with early stroke treatment: pooled analysis of ATLANTIS, ECASS, and NINDS rt-PA stroke trials. Lancet 2004;363(9411):768-774.

69. Hinds NP, Wiles CM. Assessment of swallowing and referral to speech and language therapists in acute stroke. QJM 1998;91(12):829-835.

70. Johnston KC, Li JY, Lyden PD et al. Medical and neurological complications of ischemic stroke: experience from the RANTTAS trial. RANTTAS Investigators. Stroke 1998;29(2):447-453.

71. Newell SD, Jr., Englert J, Box-Taylor A, Davis KM, Koch KE. Clinical efficiency tools improve stroke management in a rural southern health system. Stroke 1998;29(6):1092-1098.

72. Nilsson H, Ekberg O, Olsson R, Hindfelt B. Dysphagia in stroke: a prospective study of quantitative aspects of swallowing in dysphagic patients. Dysphagia 1998;13(1):32-38.

73. Pinto AN, Melo TP, Lourenco ME et al. Can a clinical classification of stroke predict complications and treatments during hospitalization? Cerebrovasc Dis 1998;8(4):204-209.

74. Schwab S, Schwarz S, Spranger M, Keller E, Bertram M, Hacke W. Moderate hypothermia in the treatment of patients with severe middle cerebral artery infarction. Stroke 1998;29(12):2461-2466.

75. Schneider D, Berrouschot J, Brandt T et al. Safety, pharmacokinetics and biological activity of enlimomab (anti-ICAM-1 antibody): an open-label, dose escalation study in patients hospitalized for acute stroke. Eur Neurol 1998;40(2):78-83.

76. Grotta J. Lubeluzole treatment of acute ischemic stroke. The US and Canadian Lubeluzole Ischemic Stroke Study Group. Stroke 1997;28(12):2338-2346.

77. Davenport RJ, Dennis MS, Wellwood I, Warlow CP. Complications After Acute Stroke. Stroke 1996;27(3):415-420.

78. Kidd D, Lawson J, Nesbitt R, MacMahon J. The natural history and clinical consequences of aspiration in acute stroke. QJM 1995;88(6):409-413.

79. Webb DJ, Fayad PB, Wilbur C, Thomas A, Brass LM. Effects of a specialized team on stroke care. The first two years of the Yale Stroke Program. Stroke 1995;26(8):1353-1357.

80. Odderson IR, McKenna BS. A model for management of patients with stroke during the acute phase. Outcome and economic implications. Stroke 1993;24(12):1823-1827.

81. Spitzer K, Thie A, Kunze K. Spontaneous subarachnoid haemorrhage: expert system for appraisal of the prognosis and computer-supported decision for therapy. J Neurol 1988;235(6):335-342.

82. Gordon C, Hewer RL, Wade DT. Dysphagia in acute stroke. Br Med J (Clin Res Ed) 1987;295(6595):411-414.

83. Przelomski MM, Roth RM, Gleckman RA, Marcus EM. Fever in the wake of a stroke. Neurology 1986;36(3):427-429.
